# EM Model-Based Device-Free Localization of Multiple Bodies †

**DOI:** 10.3390/s21051728

**Published:** 2021-03-03

**Authors:** Vittorio Rampa, Monica Nicoli, Chiara Manno, Stefano Savazzi

**Affiliations:** 1 Istituto di Elettronica, Ingegneria dell’Informazione e delle Telecomunicazioni, Consiglio Nazionale delle Ricerche, Piazza Leonardo da Vinci 32, I-20133 Milano, Italy; stefano.savazzi@ieiit.cnr.it; 2 Dipartimento di Ingegneria Gestionale, Politecnico di Milano, Piazza Leonardo da Vinci 32, I-20133 Milano, Italy; monica.nicoli@polimi.it; 3 Dipartimento di Elettronica, Informazione e Bioingegneria, Politecnico di Milano, Piazza Leonardo da Vinci 32, I-20133 Milano, Italy; chiara.manno90@gmail.com

**Keywords:** body models, diffraction models, passive localization, device-free localization, multiple targets localization, Bayesian methods, particle filters

## Abstract

In this paper, we discuss the problem of device-free localization and tracking, considering multiple bodies moving inside an area monitored by a wireless network. The presence and motion of non-instrumented subjects leave a specific footprint on the received Radio-Frequency (RF) signals by affecting the Received Signal Strength (RSS) in a way that strongly depends on people location. The paper targets specifically the modelling of the effects on the electromagnetic (EM) field, and the related inference methods. A multiple-body diffraction model is exploited to predict the impact of these bodies on the RSS field, i.e., the multi-body-induced shadowing, in the form of an extra attenuation w.r.t. the reference scenario where no targets are inside the monitored area. Unlike almost all methods available in the literature, that assume multi-body-induced shadowing to sum linearly with the number of people co-present in the monitored area, the proposed model describes also the EM effects caused by their mutual interactions. As a relevant case study, the proposed EM model is exploited to predict and evaluate the effects due to two co-located bodies inside the monitored area. The proposed real-time localization and tracking method, exploiting both average and deviation of the RSS perturbations due to the two subjects, is compared against others techniques available in the literature. Finally, some results, based on experimental RF data collected in a representative indoor environment, are presented and discussed.

## 1. Introduction

Recent research activities have shown that electromagnetic (EM) fields used for data communication can be exploited as signals of opportunity for device-free (i.e., passive) environmental radio vision [1,2,3]. Actually, the first studies concerning the effects of obstacles on the radio propagation date back to the 20s of the last century [4] at the dawn of the radio era. Since then, the works presenting and discussing the effects of the human bodies on the Radio-Frequency (RF) propagation became countless [5,6,7]. However, these research activities focused on the impairments that affect the radio communication and how to mitigate these negative effects rather than exploiting them for environmental sensing.

More recently, the first experiments [8,9] about the usage of body-induced perturbations to perform radio detection and localization have revealed that body motions leave a characteristic footprint on the Channel Quality Information (CQI) [1,10,11] or the Received Signal Strength (RSS). In fact, the received power, expressed in terms of PHY layer CQI info or MAC layer RSS values, can be exploited by networked radio devices for body detection and localization tasks. CQI/RSS perturbations and variations produced by bodies (i.e., the targets) on the RF signals are processed to extract an image of the environment and of the bodies that have induced these perturbation.

These research activities [1,8,9,10] focus on passive detection methods that, unlike active ones [12], do not require a person to wear any electronic device. Several passive methods have been proposed: the Radio Tomographic Imaging (RTI) approach has been addressed in [10] showing that the combination of multiple links and the tomographic technique enables accurate human-scale localization. More recent works focused on smart living [13,14], collaborative robotics [15,16] and assisted living applications [3,17,18], including human activity monitoring [13,19], body gesture and motion recognition [20], breathing detection [21,22], crowd density estimation [23,24], people counting [25,26,27], and Device-Free Localization (DFL) [2,22,28,29]. For DFL applications, see also the references included in [1,2], the fingerprinting approach has been widely adopted, mostly using a Bayesian framework as in [14] to increase the localization accuracy. Recently, OFDM-based MIMO DFL systems have introduced CQI phase information and multi-antenna processing [22,29], and CQI Doppler estimation [30] at the expense of a more complex processing chain w.r.t. RSS-based ones.

Despite the extensive research activities on DFL, only a few systems have been discussed in the literature for multi-targets localization [31,32,33,34,35]. The RTI method [32], based on RSS attenuation, models the multi-target RSS footprint as the linear superposition of the perturbations induced by each individual. The localization accuracy can be further improved by the simultaneous processing of RSS measurements from multiple RF channels [33]. In [24], a linear relationship between the RSS fluctuations and the number of targets in the area has been derived experimentally. A successive-cancellation algorithm has been proposed in [34] to address the problem of detection/tracking of new subjects entering/moving in the monitored area. On the other hand, RSS-based compressive sensing methods have been employed in [35] to reduce the device power consumption, still providing a reasonable localization accuracy.

The main limit of the above-mentioned methods is the linear model assumption that was drawn to be unreliable in [31] for an increasing number of targets or closely located subjects. Accurate multi-target DFL requires, in fact, to deal with simple but realistic EM-based models to account for multi-body shadowing interactions. Conventional algorithms based on fingerprinting [14], radio tomographic imaging [10], ray-tracing [36], or ad-hoc statistical [11] techniques are not suited, mostly due to an unfeasible computational burden added to include all target combinations. In addition, the linear model based on the superposition assumption becomes unrealistic for modeling the movements of crowds as shown in [31,37]. In fact, the analytical approaches, based on propagation modeling, focused on the single-target case only [38,39,40].

**Figure 1:**
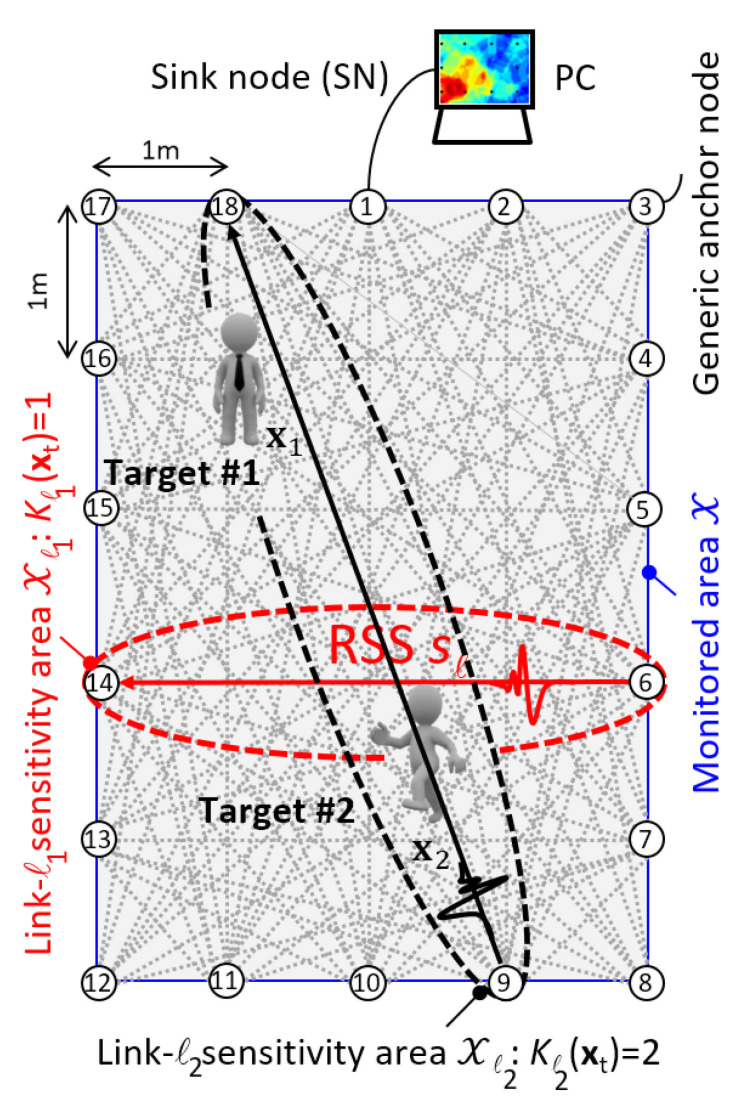
Multi-target Device-Free Localization layout: experimental scenario with N=18 fully-connected nodes and K=2 targets. The sensitivity areas for the links $\ell_{1}=\left(6,14\right)$ and $\ell_{2}=\left(9,18\right)$ are highlighted as well.

Starting from recent studies on single-target [40,41,42] and dual-target [37] human-induced fading modeling, in this paper, we propose a physical–statistical model based on the scalar diffraction theory to predict the effects [37] of two co-located targets (see Figure 1) on both RSS mean and variance. Unlike [31,40], the proposed physical–statistical model does not rely on the paraxial approximation [41] and, therefore, can be used for targets of any size placed anywhere in the monitored area and not only for small targets near the center of the link. In addition, we exploit the proposed physical–statistical model to define a novel Joint Maximum Likelihood (JML) approach that supersedes the method in [31] and a novel Bayesian dual-target DFL algorithm, that is based on the Particle Filter (PF) approach. The PF method is here validated by extensive field measurement data collected by an ad-hoc IEEE 802.15.4 wireless network. With respect to [31], the proposed Bayesian PF algorithm exploits a full two-dimensional (2D) motion model, based on both position and velocity state information, to track two independently moving targets. Finally, the localization performances of the PF method are compared against the JML [31], the successive cancellation (SC) [31], the Maximum Likelihood RTI (RTI-ML) [31] and the Least Squares RTI (RTI-LS) [10] approaches on a real indoor scenario similar to the one shown in Figure 1.

The key topic of this paper is the use and evaluation of an EM multi-body model where multipath effects are included as log-normal disturbances. Usually, EM methods are complex and time-consuming. On the other hand, the model proposed here is simple enough to be employed for real-time DFL methods and requires only a very short calibration phase. This is relevant for practical applications, as most of Machine Learning/Deep Learning (ML/DL) methods (not model-based) require a huge amount of data and a long learning phase to extract RSS-position information in scenarios with multiple targets. For details about ML/DL methods, the interested reader can take a look at [13,25,26] and, for the statistical models, at the references included in the cited surveys [2,3,6].

To summarize, the original contributions of this paper are as follows: (i) a novel dual-target EM model that accounts for small voluntary/involuntary movements of two human bodies and underpins a Bayesian framework for full dual-target localization and tracking; (ii) a procedure for the calibration of the model parameters based on field data, and the evaluation of the dual-target perturbation maps (RSS mean and standard deviations); (iii) a link selection procedure for dropping out unreliable link measurements; (iv) the design of a new family of non-Bayesian (JML, SC and ML-RTI) and Bayesian (PF) methods that exploit the new model for multi-target localization and take advantage of both average and fluctuation of RSS readings to increase the DFL accuracy; and (v) the validation and comparison of the proposed model-based methods in real indoor scenarios.

The paper is organized as follows: the problem formulation is shown in Section 2 where the Bayesian PF framework is introduced to estimate and track two targets by using a 2D motion model. The EM model for the prediction of the multiple bodies-induced shadowing is recalled and discussed in Section 3. As a practical case study, we address the problem of RSS modeling for two targets moving in a monitored area. Section 4 deals with the proposed model-based localization algorithm and its comparison with other DFL methods, used as benchmarks. Experimental results obtained in an indoor environment are shown and discussed in Section 5. Finally, some concluding remarks are drawn in Section 6.

## 2. RSS Model

We consider here the localization of *K* human targets having positions $x_{k}={\left[x_{k,1},x_{k,2}\right]}^{T}$, k=1,…,K, in a 2D area X covered by a wireless network composed of *N* anchor nodes with L≤N(N−1) radio links. The network is characterized by an undirect graph with links collected in the set $L=\left\{\ell :\;\ell =1,\ldots ,L\right\}$. The network layout for the experimental tests is sketched in Figure 1 (top): the wireless network, composed by N=18 fully connected anchor nodes, covers the area X having size 4×5 sqm where K=2 targets can move independently. Being $s_{\ell }$ the RSS in logarithmic scale (i.e., dBm) over the link ℓ∈L of length $d_{\ell }$, the objective of the DFL network is the estimation of the *K* target positions, arranged in the column vector $x={\left[x_{1}^{T}\ldots x_{K}^{T}\right]}^{T}$ of size 2K×1, by exploiting the column vector $s={\left[s_{1}\ldots s_{L}\right]}^{T}$ of size L×1 that collects the RSS observations of all links *L* over the time *t*.

According to the experimental results [1,14,40] carried out on human-induced fading in a single-target scenario (i.e., K=1), $s_{\ell }$ can be reasonably modelled as Gaussian distributed. In fact, even if other parametric distributions, e.g., Weibull and Nagakami [43], provide better results, the Gaussian approximation is widely used and provides results in good agreement with RF measures. Extending the Gaussian model to K>1 targets, we assume that the RSS $s_{\ell }$ depends on the number $K_{\ell }\left(x\right)\leq K$ of the targets that are inside the link sensitivity area $X_{\ell }$ and on their specific locations x. For further details about the link sensitivity area, the reader can refer to [40,41]. An example is sketched in Figure 1 where the $\ell_{1}$-th link identified by the node couple (6,14) is characterized by $K_{\ell_{1}}\left(x\right)=1$ since only the Target #2 falls within the link sensitivity area $X_{\ell }$ (highlighted in red in the aforementioned figure). Similar considerations apply also for the link (9,18), where it is $K_{\ell }\left(x\right)=2$ since both Target #1 and Target #2 are placed inside the link sensitivity area (highlighted in black). In general, it is:

(1) $$s_{\ell }=\left\{\begin{array}{cc} h_{0,\ell }+w_{0,\ell } & \mathrm{if}\;K_{\ell }\left(x\right)=0\\ h_{1,\ell }\left(x\right)+w_{1,\ell }\left(x\right) & \mathrm{if}\;K_{\ell }\left(x\right)>0\;. \end{array}\right.$$

If no target is in the link sensitivity area i.e., $K_{\ell }\left(x\right)=0$, then the RSS $s_{\ell }$ has a deterministic mean $h_{0,\ell }$ that accounts for path-loss and static effects due to static obstructing or scattering objects. The random term $w_{0,\ell }∼N\left(0,\sigma_{0,\ell }^{2}\right)$ models the measurement errors and the other small power fluctuations induced by variations and/or changes in the surrounding environment as well. Likewise, when one or more targets are inside the *ℓ*-th link sensitivity area $X_{\ell }$ i.e., $K_{\ell }\left(x\right)>0$, the received power $s_{\ell }$ shows an extra attenuation $\Delta h_{\ell }\left(x\right)$, w.r.t. the empty scenario term $h_{0,\ell }$, due to the obstruction(s) generated by the presence of the target(s). In addition, $s_{\ell }$ shows an increased fluctuation $\Delta \sigma_{\ell }\left(x\right)\geq 0$ w.r.t. the reference scenario term $\sigma_{0,\ell }$ due to small movements of the target(s) around the nominal position x, such as turning, change of posture, and arm and/or torso movements. Thus, the mean RSS becomes:

(2) $$h_{1,\ell }\left(x\right)=h_{0,\ell }+\Delta h_{\ell }\left(x\right)\leq h_{0,\ell }$$

while the noise term $w_{1,\ell }\left(x\right)∼N\left(0,\sigma_{1,\ell }^{2}\left(x\right)\right)$, that models the random shadowing effects, is given by:

(3) $$\sigma_{1,\ell }\left(x\right)=\sigma_{0,\ell }+\Delta \sigma_{\ell }\left(x\right)\geq \sigma_{0,\ell }.$$

The validity of (1) has been demonstrated in several experimental tests on the single-target case [14,40,44]: both the average $\Delta h_{\ell }\left(x\right)$ and the standard-deviation $\Delta \sigma_{\ell }\left(x\right)$ perturbations increase when the target is obstructing the Line-Of-Sight (LOS), especially if the target is very close to the transmitter or the receiver. Likewise, in the multi-target scenario, perturbations tend to increase with the number of targets [31,37], too. For instance, Nicoli et al. [31] shows that, for a link length $d_{\ell }=4$ m and for targets placed along the LOS path in selected landmark positions at distance of 0.5 m from the nearest one(s), the RSS mean $h_{1,\ell }\left(x\right)$ decreases of about 3.5 dB w.r.t. the empty scenario while the standard deviation $\sigma_{1,\ell }\left(x\right)$ increases of about 3 dB w.r.t. the empty scenario when the number of targets raises from K=1 up to K=7.

From the above considerations, for each *ℓ*-th link sensitivity area $X_{\ell }$, both RSS average $\left\{h_{0,\ell },h_{1,\ell }\left(x\right)\right\}$ and standard deviation $\left\{\sigma_{0,\ell },\sigma_{1,\ell }\left(x\right)\right\}$ terms of (1) provide important information about the number of targets in the link area and in turn, by combining all *L* link information, on the target locations x.

To exploit the targets footprint on both RSS average and variance terms, the knowledge of the reference maps $\left\{h_{0,\ell },\sigma_{0,\ell }\right\}$ for the empty scenario, and the perturbation maps $\left\{\Delta h_{\ell }\left(x\right),\Delta \sigma_{\ell }\left(x\right)\right\}$ is required for all links ℓ∈L, for all landmark positions, and for all targets inside the monitored area $X=\cup_{\ell }X_{\ell }$. The reference maps $\left\{h_{0,\ell },\sigma_{0,\ell }\right\}$ can be easily measured when no target is moving inside the monitored area X. For the single-target scenarios, fingerprinting maps are usually obtained by collecting RSS samples over each link while a person only is subsequently positioned over M≥K landmark points ${\left\{b_{m}\right\}}_{m=1}^{M}$ of a 2D grid covering the monitored area. However, for K>1, the evaluation of the maps $\left\{\Delta h_{\ell }\left(x\right),\Delta \sigma_{\ell }\left(x\right)\right\}$ is more critical, as it requires time-consuming ray-tracing simulations or extensive fingerprinting acquisition campaigns over different target–landmarks configurations. Given the number M≫1 of landmarks, the complexity becomes easily cumbersome even for a small number K>1 of targets present in the link sensitivity area, as the number of different targets-landmarks configurations to be explored is in the order of $O\left(M^{K}\right)$. Therefore, modeling is mandatory to simplify the calibration process.

In Rampa et al. [40,41], a closed-form analytical model has been derived for K=1 based on the diffraction theory, relating both the RSS average and standard deviation to the target location. The diffraction models were shown to fit reasonably well the attenuation effects up to K=2 targets [31,37], accounting for the joint interactions between targets. Here, we propose a novel physical–statistical model that extends Nicoli et al. [31] by exploiting a full EM model without any paraxial approximations, and Rampa et al. [37] by including small voluntary/involuntary movements of two human bodies around their nominal positions. Although not considered here, the proposed approach can be also exploited to predict the effects of a larger number of targets i.e., for K≥3.

To validate the model, in the experimental Section 5, we consider the RSS maps built by measurement field tests using the conventional fingerprinting approach [14,31]. We compare the new model against the additive perturbation maps [10,33] based on the linear superposition approximation that simplifies processing at the cost of reducing the modeling accuracy. In this case, the multi-target effects are approximated as the linear superposition of single-target terms as:

(4) $$\Delta h_{\ell }\left(x\right)=\sum_{k=1}^{K}\Delta h_{\ell }\left(x_{k}\right)$$

and

(5) $$\Delta \sigma_{\ell }^{2}\left(x\right)=\sum_{k=1}^{K}\Delta \sigma_{\ell }^{2}\left(x_{k}\right),$$

using the single-target maps $\left\{\Delta h_{\ell }\left(x_{k}\right),\Delta \sigma_{\ell }\left(x_{k}\right)\right\}$ predicted, or measured, assuming the presence of a single target only, located at position $x_{k}$.

## 3. EM-Based Model of Dual-Target RSS Perturbations

**Figure 2:**
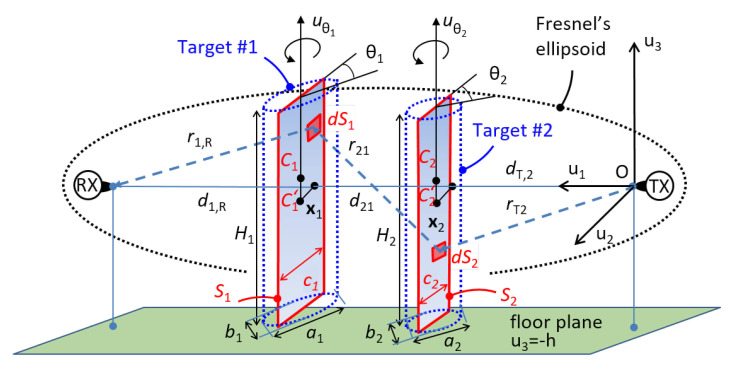
Geometry of the dual-target scenario for a single link.

In this section, we focus on a single-link scenario [31,37] with K=2 three-dimensional (3D) objects modeling the human targets, placed near the LOS path connecting the transmitter (TX) and the receiver (RX). The geometrical description of this scenario in the 3D space of coordinates $\left(u_{1},u_{2},u_{3}\right)$ is shown in Figure 2, where the TX is placed in the origin O=(0,0,0), the RX in (d,0,0), and the link is horizontally placed (along the axis $u_{1}$) at height *h* from the floor. The floor (i.e., the plane $u_{3}=-h$) does not influence the RF propagation but it is has only the purpose to support the bodies. Thus, the Fresnel’s ellipsoid [40] not only has no contact with the floor, but also with ceiling, walls or other obstacles except for the two targets moving inside the link area. These assumptions introduce the geometrical constraint $\min\left(h,\;d_{w},\;d_{c}\right)\gg \sqrt{\lambda d}/2$, where λ is the carrier wavelength, while $d_{w}$ and $d_{c}$ are the minimum distances of the link path from walls and ceiling. Each *k*-th 3D body is represented as an homogeneous and perfectly absorbing electromagnetic cylinder, with height $H_{k}$ and elliptical base with axes $a_{k},b_{k}$. Since each body can move and rotate, it shows a rectangular cross-section in the plane orthogonal to the LOS path having height $H_{k}$ and traversal size $c_{k}$ for k=1,2 with $b_{k}\leq c_{k}\leq a_{k}$.

Since 3D objects are difficult to model (and time consuming, too), bodies are approximated [37,40,41,42] as 2D knife-edge obstacles $S_{k}$, having the barycenter $C_{k}$ that has footprint $C_{k}^{{}^{\prime }}=x_{k}$ in the horizontal 2D plane containing the LOS path. To model people that stand in specific positions but might change orientation or posture, we assume, as in the single-target case [41], that each object can rotate by an angle $\theta_{k}$ around the vertical axis $u_{\theta_{k}}$, i.e., the traversal size $c_{k}$ can arbitrarily change in the range $b_{k}\leq c_{k}\leq a_{k}$. In addition, each object can also slightly change its position to $x_{k}+\Delta x_{k}$ w.r.t. the nominal position $x_{k}$. The link length *d* is equal to $d=d_{1,R}+d_{2,1}+d_{T,2}$ where $d_{1,R}$ and $d_{T,2}$ are the distances of the knife-edges $S_{1}$ and $S_{2}$ from the RX and TX, respectively. The distance between the knife-edges $S_{1}$ and $S_{2}$ is equal to $d_{2,1}$. The knife-edges are numbered in ascending order from the RX up to the TX (i.e., in Figure 2 $S_{1}$ is near the RX and $S_{2}$ near the TX, respectively).

According to Rampa et al. [37], the total electric field dE at the RX, due to the elementary areas $dS_{k}$ of both 2D knife-edges representing the targets, can be predicted by the forward propagation of the two virtual arrays of Huygens’ sources located on the obstacle planes $S_{k}^{{}^{\prime }}$ but not belonging to the obstacles $S_{k}$ themselves. However, unlike [31], that employs the paraxial approximation [40,44], the EM model adopted here refers to the full scheme presented in Rampa et al. [37]. Neglecting backward/multiple scattering effects between obstacles, and ignoring ground, walls and ceilings reflections, it is:

(6) $$dE=-E_{0}\frac{d}{r_{1,R}\;r_{2,1}\;r_{T,2}\;\lambda^{2}}\;e^{-j\;2\;\pi \;\frac{r_{T,2}\;+\;r_{2,1}\;+\;r_{1,R}\;-\;d}{\lambda }}\;dS_{1}\;dS_{2},$$

where $E_{0}$ is the free-space electric field at the RX when no bodies are present in the link area (i.e., the reference scenario). The terms $r_{1,R}$ and $r_{2,1}$ are the distances of the elementary surface $dS_{1}$ from the receiver RX and the elementary surface $d_{2}$, respectively. Likewise, the term $r_{T,2}$ is the distance of the elementary surface $dS_{2}$ from the transmitter TX. By mathematical manipulation of (6), the total received electric field *E* can be expressed as a function of the link geometry, the position and the size of the knife-edges:

(7) $$\begin{array}{cc} E= & -E_{0}\int_{S_{1}^{{}^{\prime }}}\int_{S_{2}^{{}^{\prime }}}\frac{d}{r_{1,R}\;r_{2,1}\;r_{T,2}\;\lambda^{2}}\;e^{-j\;2\;\pi \frac{r_{1,R}\;+r{\;}_{2,1}\;+\;r_{T,2}\;-\;d}{\lambda }}\;dS_{1}\;dS_{2}\\ = & -E_{0}+E_{1}+E_{2}-E_{1,2} \end{array}$$

where the term $E_{k}$:

(8) $$E_{k}=E_{0}-E_{0}\int_{S_{k}}\frac{d}{r_{k,R}\;r_{T,k}\;\lambda }e^{-j\;2\;\pi \frac{r_{T,k}\;+\;r_{k,R}\;-\;d}{\lambda }}\;dS_{k}$$

is the electric field at the RX when only the *k*-th target (i.e., k=1 or k=2) is present in the link sensitivity area [41] while the joint term $E_{k,i}$ models the interactions between the two knife-edges $S_{k}$ and $S_{i}$ with i≠k:

(9) $$E_{k,i}=E_{0}\int_{S_{k}}\int_{S_{i}}\frac{d}{r_{k,R}\;r_{i,k}\;r_{T,i}\;\lambda^{2}}\;e^{-j\;2\;\pi \frac{r_{k,R}\;+\;r_{i,k}\;+\;r_{T,i}\;-\;d}{\lambda }}\;dS_{1}\;dS_{2}.$$

Being (6)–(9) based on a forward only method, they hold only in the domain $D=\left\{\left(x_{k},y_{k}\right)\in R^{2}:\;0<x_{k}<d,\;-\infty <y_{k}<+\infty \right\}$. As shown in Rampa et al. [37], Rampa et al. [41] and sketched in Figure 1, the effect due to the target presence near the radio link practically vanishes for large but finite values of $\left|y_{k}\right|$. For each *ℓ*-th link, according to Equations (1) and (7), it is:

(10) $$\Delta h_{\ell }\left(x\right)=E_{\theta ,\Delta x}[10\log_{10}|E/E_{0}{|}^{2}]+\Delta h_{C}$$

and

(11) $$\Delta \sigma_{\ell }\left(x\right)=S_{\theta ,\Delta x}[10\log_{10}|E/E_{0}{|}^{2}]+\Delta \sigma_{C}$$

where $E_{\theta ,\Delta x}\left[\cdot \right]$ and $S_{\theta ,\Delta x}\left[\cdot \right]$ are the Expectation and Standard deviation operators w.r.t. the random vectors $\theta ={\left[\theta_{1}\;\theta_{2}\right]}^{T}$ and $\Delta x={\left[\Delta x_{1}^{T}\;\Delta x_{2}^{T}\right]}^{T}$, respectively. The terms $\Delta h_{C}$ and $\Delta \sigma_{C}$ include the multipath effects that are assumed constant in the link area and independent from the presence of the bodies.

Notice that: (i) if only the *k*-th body is present then, being $E_{k,i}=0$ and $E_{i}=E_{0}$ since the *i*-th target is absent (i≠k), then (7) reduces to the single-target case $E=E_{k}$ given in (8); (ii) it is easy to show that, if the knife-edges $S_{k}$ and $S_{i}$ have different size, then it is also $E_{k,i}\neq E_{i,k}$ since the integration domains are different. It means that, if we exchange the position of the targets, then the extra attenuation results are changed as well. On the contrary, if the targets have equal dimensions, then it is impossible to distinguish them and the final result does not change. Finally, (iii) according to (7), the total extra attenuation $10\log_{10}{\left|E/E_{0}\right|}^{2}$ w.r.t. the reference scenario, due to the combined effects of both targets, does not simplify to the superposition of the single effects (8) of each target w.r.t. the reference scenario. Thus, it is:

(12) $$10\log_{10}|E/E_{0}{|}^{2}\neq 10\log_{10}|E_{i}/E_{0}{|}^{2}+10\log_{10}{\left|E_{k}/E_{0}\right|}^{2}$$

and:

(13) $$E_{\theta ,\Delta x}[10\log_{10}|E/E_{0}{|}^{2}]\neq E_{\theta ,\Delta x}[10\log_{10}|E_{i}/E_{0}{|}^{2}]+E_{\theta ,\Delta x}[10\log_{10}|E_{k}/E_{0}{|}^{2}].$$

## 4. Multi-Target Localization Methods

We will present here a novel Bayesian method (PF) for multi-target localization based on the EM model presented in Section 3, along with a brief review of the non-Bayesian methods introduced in Nicoli et al. [31] for performance comparison. Since all localization algorithms exploit multi-link information, the equations shown in Section 3 for a single link will be used together by considering that all links lay in the horizontal plane having distance *h* w.r.t. the ground and each *ℓ*-th link has length $d_{\ell }$. Finally, the performances of the different methods will be compared in Section 5 by extensive on-field experiments. In the following sections, we will assume that the number of targets is known and equal to K=2. Extension to detection and localization of K>2 targets can be obtained by enlarging in the search for additional target positions, including states denoting the target absence, following the approach proposed in Savazzi et al. [14] for K=1.

### 4.1. Dual-Target Particle Filter Estimation (PF)

Within the Bayesian framework, the RSS model (1) can be rewritten to highlight the time dependency of the overall set of observations $s_{1:t}=\left[s_{1}\;s_{2}\cdots \;s_{t-1}\;s_{t}\right]$ collected up to time *t* by the whole network. Since the targets move in the monitored area X changing their positions $x_{t}={\left[x_{1}^{T}\left(t\right)\;x_{2}^{T}\left(t\right)\right]}^{T}$, it is:

(14) $$s_{t}=\Delta h\left(x_{t}\right)+h_{0}+w\left(x_{t}\right),$$

where the column vectors $h_{0}={\left[h_{0,1}\cdots h_{0,L}\right]}^{T}$ and $\Delta h\left(x_{t}\right)={\left[\Delta h_{1}\left(x_{t}\right)\cdots \Delta h_{L}\left(x_{t}\right)\right]}^{T}$ of size L×1 collect the empty-link mean reference and the multi-target induced deviations for all links at time *t*, respectively. The column vector $w\left(x_{t}\right)={\left[w_{1,1}\left(x_{t}\right)\cdots w_{1,L}\left(x_{t}\right)\right]}^{T}$ of size L×1 includes the Gaussian-distributed noise $w\left(x_{t}\right)∼N\left(0,Q\left(x_{t}\right)\right)$ corresponding to the log-normal shadowing effects (assumed mutually independent) having the covariance matrix $Q\left(x_{t}\right)=\mathrm{diag}\left(\sigma_{1,1}^{2}\left(x_{t}\right),\;\cdots \;,\sigma_{1,L}^{2}\left(x_{t}\right)\right)$.

According to Section 3, Equation (7) depends on the parameter set $\Theta =\{h,d,H_{1},H_{2},c_{1},c_{2},x_{t}\}\in T$ that allows us to compute all the map elements $\left\{\Delta h_{\ell }\left(x_{t}\right),\Delta \sigma_{\ell }\left(x_{t}\right)\right\}$ by using (10) and (11) as functions of the targets positions $x_{t}$ when both $\Delta h_{C}$ and $\Delta \sigma_{C}$ terms are available (or evaluated during the calibration phase as shown in Rampa et al. [40]), and the geometrical sizes $H_{k}$ and $c_{k}$ of all targets are known or estimated (see Section 5.2). The other geometrical terms *h* and $d_{\ell }$ can be easily measured for each link after the network deployment. It is worth mentioning that the RSS perturbations in (14) are set to $\Delta h_{\ell }\left(x_{t}\right)=0$ and $\Delta \sigma_{\ell }\left(x_{t}\right)=0$ outside the link sensitivity area [40] i.e., $\forall x_{t}:$ $K_{\ell }\left(x_{t}\right)=0$. If the model (7) is not available, then the proposed method reduces to the Bayesian fingerprinting approach [14] by exploiting the landmark points ${\left\{b_{m}\right\}}_{m=1}^{M}$ to evaluate for all *L* links the perturbation maps.

We exploit also the spatial continuity of the targets movements by assuming a 2D motion model to describe how the targets positions evolve over time. The constant velocity (CV) model, herein considered to describe the motion with nearly constant velocity of each target $k\in \left\{1,2\right\}$, is represented by the first-order equation [45]:

(15) $$u_{t}^{\left(k\right)}=F\;u_{t-1}^{\left(k\right)}+L\;n_{t-1}^{\left(k\right)}$$

where the 4×1 vector:

(16) $$u_{t}^{\left(k\right)}=\left[\begin{array}{c} x_{t}^{\left(k\right)}\\ v_{t}^{\left(k\right)} \end{array}\right]$$

is the joint position-velocity state for the *k*-th target while F and L are 4×4 and 4×2 motion matrices defined as

(17) $$F=\left[\begin{array}{cc} I_{2} & T\cdot I_{2}\\ 0_{2} & I_{2} \end{array}\right]$$

(18) $$L=\left[\begin{array}{c} \frac{T^{2}}{2}\cdot I_{2}\\ T\cdot I_{2} \end{array}\right].$$

$I_{2}$ and $0_{2}$ are the unitary and null matrix of size 2×2, respectively while *T* is the sampling time interval. The noise vector $n_{t}^{\left(k\right)}$ of the driving process, having size 2×1, is assumed to be Gaussian distributed $n_{t}^{\left(k\right)}∼N\left(0_{2}\;,Q_{n}\right)$ with zero mean vector $0_{2}$ and covariance matrix $Q_{n}=\mathrm{diag}\left(\sigma_{x}^{2},\sigma_{y}^{2}\right)$. Globally, the joint position-velocity state of both targets is represented by the 8×1 vector:

(19) $$u_{t}=\left[\begin{array}{c} u_{t}^{\left(1\right)}\\ u_{t}^{\left(2\right)} \end{array}\right].$$

Assuming a first order Markov prediction, the PF algorithm combines the measurements collected at time *t* with the a-priori information obtained at time t−1 to get the a-posteriori probability density function (pdf) [14] of the target location $x_{t}$ at time *t*. The a-priori pdf $p\left(x_{t}|s_{1:t-1}\right)$ is obtained by the prediction step by using the a-posteriori pdf $p\left(x_{t-1}|s_{1:t-1}\right)$ at time t−1 and the transition pdf $p\left(x_{t}|x_{t-1}\;\right)$ of the motion model according to the Chapman–Kolmogorov equation:

(20) $$\begin{array}{c} p\left(x_{t}|s_{1:t-1}\right)=\int p\left(x_{t}|x_{t-1}\right)\;p\left(x_{t-1}|s_{1:t-1}\right)\;dx_{t-1}. \end{array}$$

The a-posteriori pdf $p\left(x_{t}|s_{1:t}\right)$ is computed in the update step by using the Bayes theorem with the conditional probability $p\left(s_{t}|x_{t}\right)$ evaluated according to the perturbation maps and the a-priori pdf (20):

(21) $$p\left(x_{t}|s_{1:t}\right)=\frac{p\left(s_{t}|x_{t}\right)\;p\left(x_{t}|s_{1:t-1}\right)}{p\left(s_{t}|s_{1:t-1}\right)}.$$

Evaluation of the conditional probability $p\left(s_{t}|x_{t}\right)$ is performed as shown in (28) according to the perturbation maps obtained by exploiting the physical–statistical model (14) or the measurement one (1). Finally, the position estimate is computed as the Minimum Mean Squared Error (MMSE):

(22) $${\hat{x}}_{t|t}=\int x_{t}\;p\left(x_{t}|s_{1:t}\right)dx_{t}.$$

To avoid the computational complexity of the grid-based search in the detection area X, we adopted here the Particle Filtering method [46]. This is a Monte Carlo sequential approach, that represents the a-priori pdf with a discrete set of particles:

(23) $$p\left(x_{t}|s_{1:t-1}\right)∼\frac{1}{N_{p}}\sum_{n=1}^{N_{p}}\delta \left(x_{t}-x_{t,n}\right),$$

where the $N_{p}$ particles, $x_{t,n}={\left[x_{1,n}^{T}\left(t\right)\;\cdots \;x_{K,n}^{T}\left(t\right)\right]}^{T}$, describing the Cartesian coordinates ${\left\{x_{t,n}\right\}}_{n=1}^{N_{p}}$, are independent identically distributed (i.i.d.) random variables with pdf $x_{t}∼p\left(x_{t}|s_{1:t-1}\right)$. Equation (21) is rewritten as:

(24) $$\begin{array}{c} p\left(x_{t}|s_{1:t}\right)∼\frac{1}{N_{p}}\sum_{n=1}^{N_{p}}p\left(s_{t}|x_{t}\right)\delta \left(x_{t}-x_{t,n}\right). \end{array}$$

The conditional pdf $p\left(s_{t}|x_{t}\right)$ is defined as:

(25) $$q_{t,n}=\frac{{\tilde{q}}_{t,n}}{\sum_{n=1}^{N_{p}}{\tilde{q}}_{t,n}},$$

by normalizing the term ${\tilde{q}}_{t,n}=\frac{p\left(s_{t}|x_{t}=x_{t,n}\right)}{N_{p}}$. Therefore, (24) simplifies to:

(26) $$p\left(x_{t}|s_{1:t}\right)=\sum_{n=1}^{N_{p}}q_{t,n}\delta \left(x_{t}-x_{t,n}\right).$$

The estimated locations of the subjects is found by applying the MMSE criterion as:

(27) $${\hat{x}}_{t|t}=E_{t}\left[x_{t}|s_{1:t}\right]=\sum_{n=1}^{N_{p}}q_{t,n}x_{t,n}.$$

At time step t+1 a new set of particles must be generated ${\left\{x_{t,n},\;q_{t,n}\right\}}_{n=1}^{N_{p}}$ using the resample and propagation steps [14]. Finally, the initial probability $p\left(x_{0}|s_{0}\right)=p_{0}\left(x_{0}\right)$ at time t=0 can be chosen according to the available information about the targets position $x_{0}$. For instance, if the target entrance points are constrained to be in *G* fixed positions $x_{0}^{\left(g\right)}={\left[x_{1}^{\left(g\right)}\;x_{2}^{\left(g\right)}\right]}^{T}$, then for g=1,...,G, it is $p_{0}\left(x_{k}\right)=\delta \left(x_{k}-x_{0}^{\left(g\right)}\right)/G$ where δ(·) is the Dirac delta function.

### 4.2. Joint-ML Estimation (JML)

Unlike PF, the joint multi-target ML estimation algorithm (JML) obtains the target positions in a snapshot manner as ${\hat{x}}_{t}=\arg\max_{x_{t}\in X}\Lambda \left(s_{t}|x_{t}\right)$ using only the information available at time *t*. Given the Gaussian assumption for $w(x_{t})$, the log-likelihood function $\ln p\left(s_{t}|x_{t}\right)$ is computed as:

(28) $$\Lambda \left(s_{t}|x_{t}\right)=-\ln\left|Q\left(x_{t}\right)\right|-{∥s_{t}-\Delta h\left(x_{t}\right)-h_{0}∥}_{Q^{-1}\left(x_{t}\right)}^{2},$$

where |·| denotes the determinant and ${∥a∥}_{C}^{2}=$ $a^{T}C\;a$ is the weighted squared norm of a vector a by the matrix C. The perturbation maps ${\left\{\Delta h_{\ell }\left(x_{t}\right),\Delta \sigma_{\ell }\left(x_{t}\right)\right\}}_{\ell =1}^{L}$ can be model-based or measured in some landmark positions during the initial calibration phase according to the fingerprinting approach (see Section 4.1).

### 4.3. Successive Cancellation Estimation (SC)

The successive cancellation method is an iterative algorithm that estimates the position of one target at a time. Assuming the model (14) with K=1 target in the monitored area, the SC algorithm estimates the position of the first target, namely ${\hat{x}}_{1,t}$, using the ML approach using only the single-target perturbation maps:

(29) $${\hat{x}}_{1,t}=\arg\max_{x_{1,t}\in X}\;\Lambda \left(s_{t}|x_{1,t}\right).$$

The contribution of the first target estimate ${\hat{x}}_{1,t}$ is then taken into account for the estimation of the second target, as:

(30) $${\hat{x}}_{2,t}=\arg\max_{x_{2,t}\in X}\;\Lambda \left(s_{t}|x={\left[{\hat{x}}_{1,t}^{T}\;x_{2,t}^{T}\right]}^{T}\right),$$

where the likelihood Λ is evaluated assuming the perturbation maps $\Delta h_{\ell }\left({\hat{x}}_{1,t},x_{2,t}\right)=\Delta h_{\ell }\left({\hat{x}}_{1,t}\right)+\Delta h_{\ell }\left(x_{2,t}|{\hat{x}}_{1,t}\right)$ and $\Delta \sigma_{\ell }^{2}\left({\hat{x}}_{1,t},x_{2,t}\right)=\Delta \sigma_{\ell }^{2}\left({\hat{x}}_{1,t}\right)+\Delta \sigma_{\ell }^{2}\left(x_{2,t}|{\hat{x}}_{1,t}\right)$, where the terms $\Delta h_{\ell }\left(x_{2,t}|{\hat{x}}_{1,t}\right)$ and $\Delta \sigma_{\ell }^{2}\left(x_{2,t}|{\hat{x}}_{1,t}\right)$ denote the contributions of the second target when the first one is located in ${\hat{x}}_{1,t}$. All these maps are computed using the diffraction-based model. The procedure is then repeated till the *K*-th user. It is apparent the linearity assumption adopted for the evaluation of $\Delta h_{\ell }$ (see the remarks in Section 3).

### 4.4. Radio Tomographic Imaging Estimation (RTI)

The tomographic imaging approach herein proposed extends the method [10] originally accounting for average RSS information only. The method derived below jointly accounts for the impact of the targets on the RSS average and RSS fluctuations (14) by estimating a motion image of the area X that captures the power variations w.r.t. the reference scenario. The monitored area X is divided into *M* voxels centered around the landmark locations ${\left\{b_{m}\right\}}_{m=1}^{M}$, while the estimated vector $v={\left[v_{1}\cdots v_{M}\right]}^{T}\in V=\left\{\forall m=1,...,M:v_{m}=0,1\right\}$ of size M×1 (i.e., the motion image) indicates whether a moving target is observed, or not, in the *m*-th voxel as $v_{m}=1$ or $v_{m}=0$, respectively. For sparse motion and assuming linearity in the power measurements, the RSS terms (1) can be approximated as the sum of the contributions generated by all occupied voxels:

(31) $$s_{\ell }=\sum_{m=1}^{M}\Delta h_{\ell ,m}v_{m}+h_{0,\ell }+w_{\ell },$$

where $\Delta h_{\ell ,m}=\Delta h_{\ell }\left(b_{m}\right)$ is the extra attenuation due to a target that occupies the *m*-th voxel. The corresponding power fluctuation term is modeled as $w_{\ell }∼N\left(0,\sigma_{\ell }^{2}\left(v\right)\right)$ with $\sigma_{\ell }\left(v\right)=\sigma_{0,\ell }+\Delta \sigma_{\ell }$ and $\Delta \sigma_{\ell }=\sqrt{\sum_{m=1}^{M}v_{m}\Delta \sigma_{\ell ,m}^{2}}$ where $\Delta \sigma_{\ell ,m}=\Delta \sigma_{\ell }\left(b_{m}\right)$ is the contribution due to the target located in voxel *m*, i.e., $x_{t}=b_{m}$. Considering all links, Equation (14) can be rewritten at time *t* as:

(32) $$s_{t}=\Delta H\cdot v_{t}+h_{0}+w_{t},$$

where the matrix ΔH$=[\Delta h_{\ell ,m}]$ of size L×M collects perturbations for all links and voxels, $v_{t}$ is $v_{t}=v\left(t\right)$, and $w_{t}=w\left(v_{t}\right)∼N\left(0,Q\left(v_{t}\right)\right)$ has covariance $Q\left(v_{t}\right)=\mathrm{diag}(\sigma_{1}^{2}\left(v_{t}\right),\cdots \;,$$\sigma_{L}^{2}\left(v_{t}\right))$ depending on the target locations.

Unlike the least squares (LS) radio-tomographic approach (RTI-LS) that employs the closed-form LS estimation ${\hat{v}}_{t}=$$\Delta H^{†}(s_{t}-h_{0})$ by using extra attenuation information only [10], the proposed RTI-ML estimate is obtained as:

(33) $${\hat{v}}_{t}=\arg\max_{v_{t}\in V}\Lambda \left(s_{t}|v_{t}\right)$$

with

(34) $$\Lambda \left(s_{t}|v_{t}\right)=-\ln|Q\left(v_{t}\right)\left|-|\right|s_{t}-\Delta Hv_{t}-h_{0}{\left|\right|}_{Q{\left(v_{t}\right)}^{-1}}^{2}.$$

The targets positions are then estimated as the *K* voxels associated with the maximum values in ${\hat{v}}_{t}$. The perturbation maps ${\left\{\Delta h_{\ell }\left(b_{m}\right),\Delta \sigma_{\ell }\left(b_{m}\right)\right\}}_{\ell =1}^{L}$ are measured in the landmark positions ${\left\{b_{m}\right\}}_{m=1}^{M}$ during the initial calibration phase according to the fingerprinting approach.

## 5. Experimental Results

In this section, we describe the network setup used for indoor measurements during an experimental on-field campaign. Some indoor and outdoor measurements have been carried but we focus only on the indoor ones as they are the most challenging. We first discuss the dual-body model calibration scheme adopted to estimate the target parameters Θ that are employed in the PF localization algorithm. Then, we compare the results obtained with the proposed method against other model-based and non-model-based DFL algorithms.

### 5.1. Device Configuration and Network Setup

**Figure 3:**
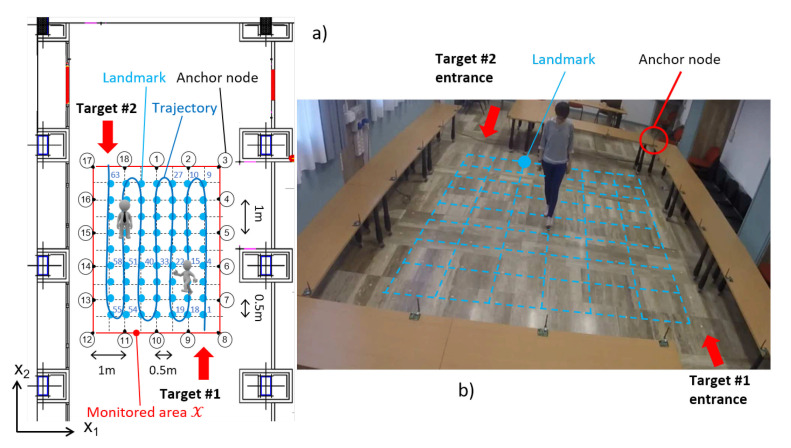
Network layout for double-target localization tests: (**a**): the targets (grey) enter the monitored area X by using G=2 different entrances and move along the trajectory (dark blue) in opposite directions. The landmark points (light blue) are labelled from 1 up to M=63; (**b**): photo of the network layout during single-target data acquisition.

Experimental validation trials have been carried out in a large hall at DEIB, Politecnico di Milano, where two people (female, about 1.65 m, and 50 kg each) freely moves. A fully connected mesh network of N=18 anchor nodes (and L=306 links) was uniformly deployed along the perimeter of a 4 m × 5 m area, spaced 1 m apart and placed on stands h=0.7 m high (see Figure 3). Each anchor node is equipped with a NXP JN5148 System-on-chip (SoC) wireless micro-controller, that enables time-slotted transmissions within the 2.4 GHz band according to the IEEE 802.15.4 standard, and other ancillary interface components [47]. The RSS dynamic range of the SoC built-in receiver is equal to 75 dB with a minimum sensitivity of −95 dBm while the transmit power is about 0 dBm. All wireless nodes employ omnidirectional vertically-polarized antennas with 2 dBi gain. Node-related noise effects are in line with what reported in Chen and Terzis [48] for other IEEE 802.15.4-compliant devices.

A modified MAC layer [1], defined on top of the beacon-enabled mode of the standard IEEE 802.15.4, allows time-slotted communications between nodes while a specific API (Application Programming Interface) has been designed to retrieve the RSS values from each anchor node. The Network Coordinator (NC), see Figure 1, creates a 60 ms custom periodic beacon frame that is transmitted at the beginning of each super-frame [14]. The beacon frame allows every wireless node to synchronize the RSS acquisition: therefore, the RSS values are synchronously sampled and time-stamped every 60 ms over all links. The anchor node 1 acts as a sink node (SN), collecting data from all nodes. Then, through a USB connection, the SN forwards the RSS information, to an external PC where RSS data and target movements are synchronized using a video camera. Finally, RSS values are processed and visualized.

The network layout adopted for double-target localization is sketched in Figure 3. In this picture, two targets follow the same trajectory (i.e., the light blue snake-like path) but in opposite directions: Target #1 enters from the bottom-right corner entrance located near nodes 8 and 9 while Target #2 gets into the area by accessing the top-left entrance located between nodes 17 and 18. Thus, the number of entrance points is G=2. A regular landmark grid (i.e., the blue dots in Figure 3) is used for fingerprinting and analytical evaluation of the perturbation maps, with M=63 landmark points spaced apart by 0.5 m along both vertical and horizontal axes. Landmark points are labelled from m=1 (bottom-right) up to m=M (top-left). The single-target fingerprint maps of RSS mean and standard deviation (see Section 5.4) are computed as in Savazzi et al. [14] by averaging the RSS samples collected over a period of 20 s. During this period, the target is moving/wandering randomly around the nominal landmark position.

### 5.2. Dual-Body Model Calibration

Calibration of the dual-target diffraction model (7) requires the estimation of the parameters $\hat{\Theta }$ from the training RSS dataset. Here, the body-induced RSS perturbations have been measured on the link connecting nodes 6 and 14, namely the link $\ell_{1}=\left(6,14\right)$ sketched in Figure 1, for all possible position combinations of two targets along some landmark positions. These landmark positions belong to the LOS path of the link $\ell_{1}$ and are identified (see also Figure 3) by the indexes of the set $M_{6,14}=\left\{m\in Z^{+}:m=4,15,22,33,40,51,58\right\}$.

Estimation of the EM model parameters for K=2 targets requires a limited calibration phase that is performed on a few landmarks (M=7), and not on all landmarks (M=63) as needed by the full fingerprinting approach. Moreover, to reduce the calibration phase, both targets are assumed to be identical. Thus, the target size parameters are estimated using the LS approach applied to all 21 different dual-target combinations $c\left(x_{1},x_{2}\right)$ instead of the full 42 combinations required using different size targets. It is:

(35) $$\begin{array}{c} \hat{\Theta }=\arg\min_{\Theta \in T}\{\sum_{c\in C}(\Delta h_{\ell }\left(c\left(x_{1},x_{2}\right)\right)+\\ \left.{\left.-E_{\theta ,\Delta x}\left[10\log_{10}\left\{{\left|E\left(c\left(x_{1},x_{2}\right),\Theta \right)/E_{0}\right|}^{2}\right\}\right]\right)}^{2}\right\} \end{array}$$

where $\Delta h_{\ell }\left(c\left(x_{1},x_{2}\right)\right)$ are the measurements and $E_{\theta ,\Delta x}\left[10\log_{10}\left\{{\left|E\left(c\left(x_{1},x_{2}\right),\Theta \right)/E_{0}\right|}^{2}\right\}\right]$ the values predicted by the EM model for the given dual-target combination $c\left(x_{1},x_{2}\right)$.

Similarly to what done in Rampa et al. [41], Rampa et al. [49], for each spatial placement of the two targets, the RSS moments $E_{\theta ,\Delta x}[10\log_{10}|E/E_{0}{|}^{2}]$ and $S_{\theta ,\Delta x}[10\log_{10}|E/E_{0}{|}^{2}]$ have been evaluated by averaging over $10^{4}$ realizations of uniform azimuth rotations θ∼U(−π,π) and uniform location uncertainties Δx∼U(−B/2,+B/2) within a bin of size B×B with B=20 cm centered around the nominal landmark points ${\left\{b_{m}\right\}}_{m\in M_{6,14}}$. Both terms θ and Δx simulate small voluntary/involuntary body movements Δx around the landmark positions. According to (35), the diffraction-based parameter estimates $\hat{\Theta }$, employed in the experimental tests of Section 5.4, correspond to two targets (female, about 1.65 m, and 50 kg each) having dimensions $a_{1}=a_{2}=41$ cm, $b_{1}=b_{2}=12$ cm, and $H_{1}=H_{2}=150$ cm. With respect to the target(s) size, it has to be noted that both knife-edge height and traversal size are loosely related to the nominal physical size of the target(s) [37,41] as these optimized values refer only to the calibration procedure previously described.

Relevant multipath effects $\Delta h_{C},\;\Delta \sigma_{C}$ have been observed in the considered indoor environment, especially due to the vertical metal foils covering the pillars along the left and right sides of the room (i.e., near anchor nodes 3,6,14 and 17) sketched in Figure 3. These effects are ignored by the diffraction model (7), while they are accounted for in the fingerprinting (i.e., measured) maps as concern the single target impact (i.e., not the interaction between the two targets).

**Figure 4:**
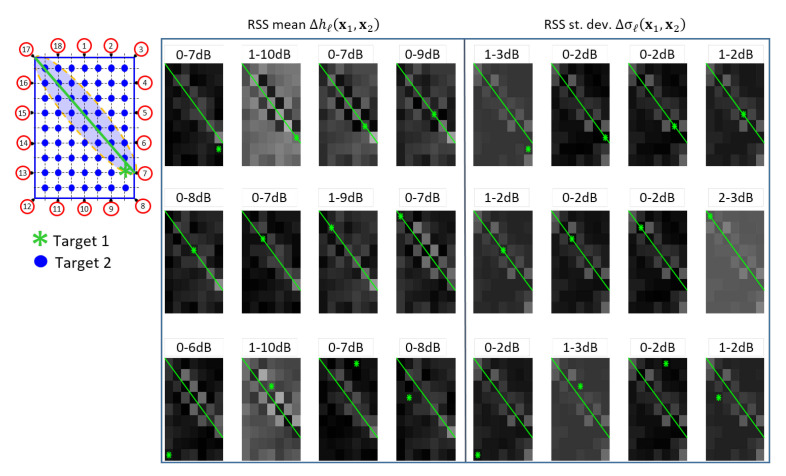
Example of perturbation maps of additive fingerprinting. The maps of size 9×7 pixels are associated to the specific *ℓ*-th link (green line) corresponding to the nodes (7,17) (see the scenario on the left). The green asterisks represent the fixed locations $x_{1}$ of Target #1 while the Target #2 moves over all landmark points $x_{2}\in {\left\{b_{m}\right\}}_{m=1}^{63}$ (blue). Each pixel represents in gray scale the value of the measured extra attenuation ${\left\{\Delta h_{\ell }\left(x_{1},x_{2}\right)\right\}}_{m=1}^{63}$ from 0 dB (black) to 15 dB (white) and that of the measured fluctuations ${\left\{\Delta \sigma_{\ell }\left(x_{1},x_{2}\right)\right\}}_{m=1}^{63}$ from 0 dB (black) to 7 dB (white). On the top of each maps, the minimum and maximum values of perturbations are shown.

**Figure 5:**
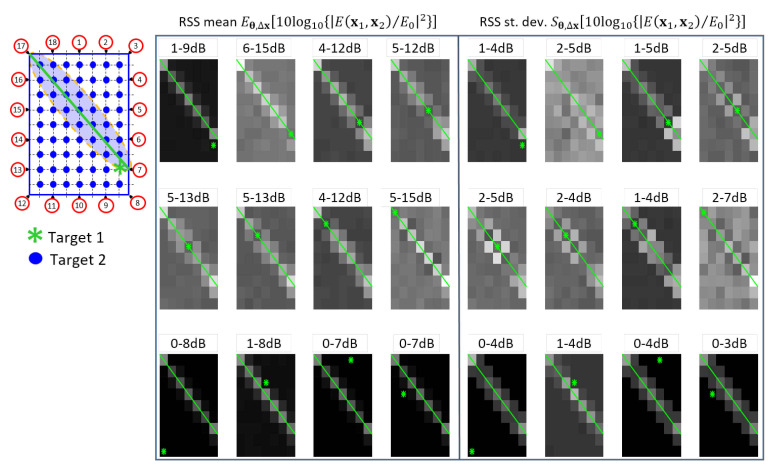
Example of perturbation maps of the dual-target diffraction model. The maps of size 9×7 pixels are associated to the specific *ℓ*-th link (green line) corresponding to the nodes (7,17) (see the scenario on the left). The green asterisks represent the fixed locations $x_{1}$ of Target #1 while the Target #2 moves over all landmark points $x_{2}\in {\left\{b_{m}\right\}}_{m=1}^{63}$ (blue). Each pixel represents in gray scale the value of the predicted extra attenuation $E_{\theta ,\Delta x}\left[10\log_{10}\left\{{\left|E\left(x_{1},x_{2}\right)/E_{0}\right|}^{2}\right\}\right]$ from 0 dB (black) to 15 dB (white) and that of the predicted extra fluctuations $S_{\theta ,\Delta x}\left[10\log_{10}\left\{{\left|E\left(x_{1},x_{2}\right)/E_{0}\right|}^{2}\right\}\right]$ from 0 dB (black) to 7 dB (white). On the top of each maps, the minimum and maximum values of perturbations are shown.

The examples of Figure 4 and Figure 5 show the additive fingerprinting and the diffraction-based perturbation maps, respectively. For a selected link (between devices 7 and 17), the figures show the perturbation measured when Target #1 is placed in the position denoted by the green star, while Target #2 moves all over the grid points of the considered area. In these maps, each pixel indicates the value of the RSS attenuation and standard deviation due to the presence of the two targets. The results show that when Target #1 is far from the LOS link (green line), these values are very similar to those experienced when only Target #2 is in the LOS area [14]. On the other hand, higher variations are tested when target 1 is in LOS and the other one is in the sensitivity area of the link. In both cases, the figures show a clear sensitivity to the presence of both targets along the links. Furthermore, as shown also in Figure 5 of reference [31], the comparison between model (7) and measurements shows that the diffraction-based model better approximates the double-target shadowing with respect to the simplified additive approach, despite the mismodeling due to multipath effects that are not included in the diffraction model (7).

As mentioned before, the dual-body model calibration requires only a few measurements taken in a subset of the landmarks. It is worth noticing that the usage of the fingerprinting methods for the dual-target scenario would require the full M(M−1)=3906 target-landmark measurement combination set: this is impractical in almost all DFL scenarios. To solve this problem, we propose here to adopt a model-based approach within the PF framework.

### 5.3. Link Selection Procedure

We recall that the EM diffraction model (7) does not consider propagation phenomena such as reflections and refractions from the floor, ceiling, walls and other massive objects that are present in the monitored area. These effects are included as noisy contributions in the Gaussian terms of (1). Depending on link locations, these noisy effects are observable as introducing outliers in the RF measurements [14]. To handle these cases and avoid performance degradation due to model mismatching, we propose here a novel method for automatic detection of the unreliable links. To this aim, by exploiting the Gaussian assumptions, we define, for each *m*-th landmark, the following reliability metric $\eta_{\ell }\left(b_{m}\right)>0$ as:

(36) $$\eta_{\ell }\left(b_{m}\right)=\frac{{∥s_{\ell ,m}-h_{\ell }\left(b_{m}\right)∥}^{2}}{\sigma_{\ell }^{2}\left(b_{m}\right)}.$$

Initially, for each *m*-th landmark, all *L* links are sorted in the ascending order ${\left\{\eta_{\ell }\left(b_{m}\right)\right\}}_{\ell =1}^{L}$; then a link *ℓ* is classified as unreliable (i.e., $U_{\ell ,m}=1$) or reliable (i.e., $U_{\ell ,m}=0$) for the *m*-th landmark by exploiting the definition $U_{\ell ,m}=I\left(\eta_{\ell }\left(b_{m}\right)\geq \gamma_{m}\right)$ where $I\left(\cdot \right)$ is the indicator function and $\gamma_{m}$ a location-dependent threshold. If a link $\ell^{{}^{\prime }}$ is labelled as unreliable more than Ω times over the entire landmark set i.e., $\sum_{m=1}^{M}U_{\ell^{{}^{\prime }},m}>\Omega$, then it is discarded from the link set L. The remaining set $\bar{L}=L-\left\{\ell^{{}^{\prime }}:\;\sum_{m=1}^{M}U_{\ell^{{}^{\prime }},m}>\Omega \right\}$ is then exploited for the DFL algorithms described in Section 4. For each *m*-th landmark, the threshold $\gamma_{m}$ is computed as $\gamma_{m}=\eta_{\bar{\ell }+1}\left(b_{m}\right)$ where $\bar{\ell }\in L$ is the smaller index such that:

(37) $$\frac{\sum_{i=1}^{\bar{\ell }}\eta_{i}\left(b_{m}\right)}{\bar{\ell }\;\eta_{\bar{\ell }+1}\left(b_{m}\right)}\leq 1-\delta$$

where δ is a chosen positive constant (0<δ<1) valid for all landmark points. The constants δ and Ω are scenario-dependent parameters: a small value of the parameter δ implies that only a few links may be discarded, while the opposite happens when δ is close to one. On the contrary, the parameter 1<Ω≤M sets a threshold on the fraction (Ω/M) of measurement landmarks that must be considered not to discard a link.

**Figure 6:**
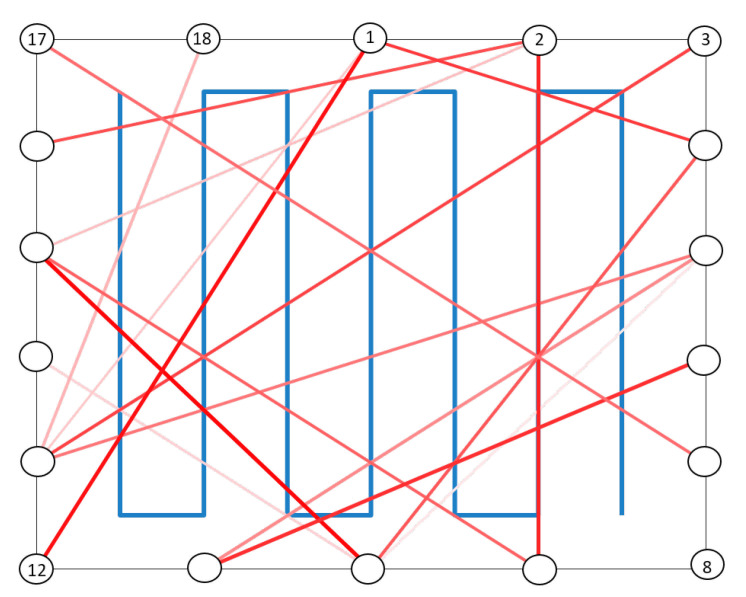
Graphical example of the network links to be deleted by the link selection procedure of the PF algorithms for the layout of Figure 1 and Figure 3 according to the procedure parameters Ω=25 and δ=0.1. Color intensity denotes the link unreliability, with deep red indicating highly unreliable links and light red less critical ones.

The example of Figure 6 for the PF algorithms of Section 4.1 shows the deleted links for the network layout of Figure 1 and Figure 3. The color intensity quantifies the unreliability level of each deleted link, namely the number of times each link is discarded: strong colored links refer to links that have been discarded a number of times much greater than the threshold Ω=25 while lightly colored links indicate that these links have been discarded only a few times over the threshold. This example assumes that the coefficient δ is set to δ=0.1.

The link selection procedure has been adopted in the early evaluation stages during the algorithm testing since it became apparent that some links were not informative at all. According to the evaluation of the model-based PF algorithm shown in Section 5.4, the degraded RMSE in the unoptimized case without the link selection procedure, for the selected layout and trajectory considered in Figure 3, is in the order of 1.3 m–1.6 m depending on the filtering parameters.

The link selection procedure described here is based on an initial calibration phase that requires only a single-target covering M=63 landmarks. It is worth noticing that the number of calibration measurements scales linearly with the landmark number *M*, in line with almost all single-target DFL approaches.

### 5.4. Algorithm Evaluation

As far as the comparison with other methods is concerned, it is not fair to directly compare single-target and multi-target methods due to the interfering effects of one target to the other ones(s) as well as the different layout and trajectory configurations adopted by the each work. In fact, as pointed out in Savazzi et al. [14] for single-target methods, the RMSE depends on the network layout and the specific trajectory adopted. In addition, as shown in [1,40] for the single-target cases, the location accuracy depends also on the number of anchor nodes and the geometry of the wireless links. Similar considerations can be also done for multi-target scenarios [31,37]. In addition to the mutual influence between targets, the target association problem is also more important in multi-target scenarios, as shown e.g., in Nicoli et al. [46], than in single-target ones. This means that the RMSE is not only location- and layout-dependent but its value depends also on the number of the tracked targets. Therefore, it is very difficult to directly compare different configurations, scenarios and number of targets.

For these reasons, in this section, we will compare the performances of the PF algorithm against other multi-target methods in the selected dual-target scenario. In addition, since the key topic of this paper is the proposal of an EM model for attenuation prediction, we will use and compare different types of perturbation maps. More specifically, in the calculation of the location estimates, we will exploit not only the Diffraction Model (DM) of Section 3 but also the Additive Fingerprinting (AF) perturbations maps $\left\{\Delta h_{\ell }\left(x_{t}\right),\;\Delta \sigma_{\ell }\left(x_{t}\right)\right\}$ of Section 2 as benchmark. To improve the localization accuracy of the PF algorithm, we will also employ the link selection criterion based on link reliability that was previously presented in Section 5.3.

**Figure 7:**
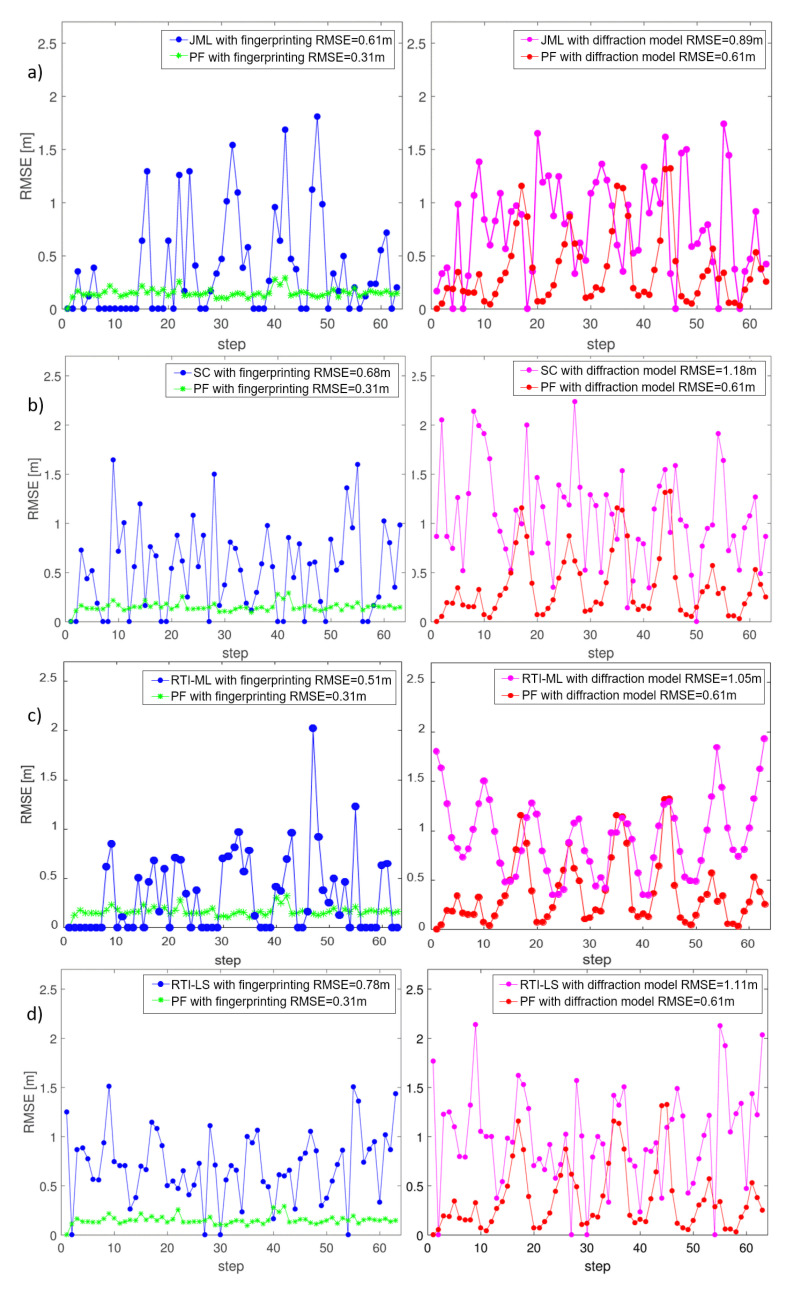
From top to bottom: (**a**) location accuracy comparison along the trajectory steps for the Joint Maximum Likelihood (JML); (**b**) successive cancellation (SC); (**c**) Radio Tomographic Imaging (RTI)-Maximum Likelihood (ML); and (**d**) RTI-Least Squares (LS) w.r.t. the Particle Filter (PF) algorithm, respectively. Both additive fingerprinting (left sub-images) and diffraction-based (right sub-images) perturbation maps are evaluated. The RMSE averaged over the whole trajectory steps is indicated on the top of each sub-figure.

Since the DFL algorithms assume that each position can be visited by only one target at a time, we expect to observe a significant localization error for snapshot (non-Bayesian) methods, such as JML, SC, RTI-ML and RTI-LS, w.r.t. the PF method, when the two targets are close to each other and when they are in the central area where the two trajectories intersect. The RTI-LS method with fingerprinting is described in Wilson and Patwari [10]; it has been adapted here to embed the diffraction model (see Figure 7d).

We assume the following PF parameters: $N_{p}=600$, Ω=25 unreliable links limit, δ=0.1, and step time T=360 ms. Moreover, the covariance matrix $Q_{n}$ of the motion model for both targets has standard deviation values set to $\sigma_{x}=\sigma_{y}=0.1$ m/step${}^{2}$ equivalent to $\sigma_{x}=\sigma_{y}=0.77$ m/s${}^{2}$. The physical–statistical model parameters (see Section 5.2) are: bin size B=20 cm, target dimensions $a_{1}=a_{2}=41$ cm, $b_{1}=b_{2}=12$ cm, $H_{1}=H_{2}=150$ cm, and link height h=70 cm. These values are summarized in Table 1.

Assuming *K* known, the accuracy of the considered algorithms namely PF, JML, SC, RTI-LS and RT-ML is evaluated, using both AF and DM perturbation maps, in Table 2. The location accuracy is evaluated in terms of the location Root Mean Squared Error (RMSE) values:

(38) $$\mathrm{RMSE}\left(x\right)=\sqrt{\frac{1}{MK}\sum_{m=1}^{M}\sum_{k=1}^{K}{∥{\hat{x}}_{k,m}-x_{k,m}∥}^{2}}$$

by averaging the location performances of each method over the M=63 positions $x_{k,m}=b_{m}$ of the considered trajectory and over the K=2 targets. The *k*-th target position estimate ${\hat{x}}_{k}\left(t\right)={\hat{x}}_{k,m}$ is computed by using the RSS observations collected over 4 s corresponding to the *m*-th landmark $b_{m}$. The location accuracy of each method is also shown for each landmark step of the trajectory.

For the simulation of the JML and PF methods based on fingerprinting maps, we used the measured single-target perturbations extended to the dual-target case by using the linear superposition model (4) and (5). Similarly, for the dual-target SC and RTI methods with fingerprinting, we used the single-target maps.

The accuracy of the multi-target localization methods is also assessed for each landmark steps of the target trajectories in Figure 7 where the considered algorithms JML, SC, RTI-ML and RT-LS are compared against the PF methods.

The comparisons of the accuracy of multi-target location for PF and the JML algorithms are assessed in Figure 7a. We see, in the first plot (left), that when we used the fingerprinting perturbation maps, the PF algorithm has a low constant error trend with respect to the JML one. In fact, the RMSE of the PF method averaged over the whole trajectory is half of the averaged JML RMSE. The second plot (right) shows the performance of JML and PF when the perturbation maps are given by a diffraction-based model. We also notice that the PF algorithm improves the localization performances with respect to the JML. Figure 7b,c show the performances of SC and RTI-ML with respect to PF, respectively. In these cases, the RMSE of the PF algorithm is lower than the ones of the other algorithms. The performances of the RTI-LS method, already available in the literature [10], are also shown in Figure 7d (left).

The JML, SC, RTI-ML and RTI-LS algorithms, that employ the analytical RSS model (right), have lower accuracy compared to the corresponding fingerprinting approach (left). However, the results of the PF algorithm with the diffraction-based model show that the localization accuracy is better, or similar, to the ones of the other fingerprinting-based methods. Finally, it has to be noted that the PF method based on the diffraction model does not require the full fingerprinting steps thus dramatically reducing the initial calibration phase.

**Figure 8:**
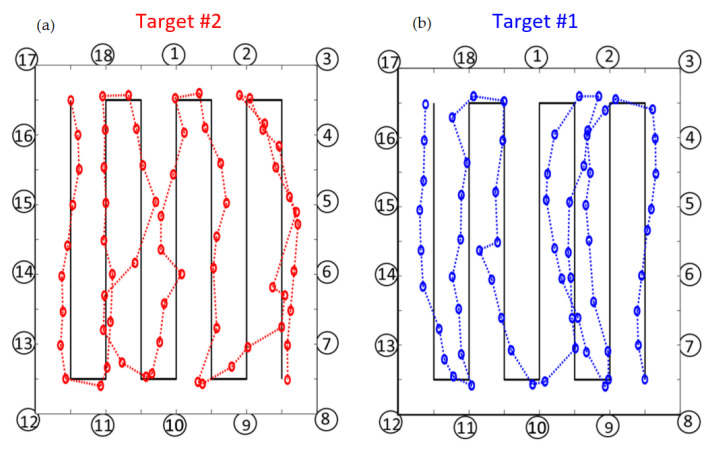
Trajectory of the Target #1 (**b**) and the Target #2 (**a**) with the use of dynamic position-velocity model in case of diffraction-based model perturbation maps.

Figure 8 shows the trajectories of Target #1 vs. Target #2 for the PF method. It is apparent that in the turn areas there are sudden changes of speed that can introduce location errors. Moreover, in the central part of the layout there are larger errors due to the fact that the two trajectories intersect each other and the targets may become indistinguishable. In spite of the challenging indoor conditions with two close-by users walking over complex trajectories with several u-turns, the proposed EM-model assisted PF DFL is able to discriminate and reconstruct the two trajectories with an average accuracy of 0.61 m. This is obtained by calibrating only few parameters of the EM physical–statistical model without the need of an extensive (unfeasible with 2 targets) full fingerprinting calibration procedure.

Finally, it has to be noted that the RMSE of the dual-target PF method with fingerprinting (i.e., 31 cm) is about 2–3 times the best single-target algorithms available in the literature [14,29,39]. The higher error is due to the complex tracing conditions with two nearby people and to the linear superposition assumption used to build the fingerprinting maps from single-target measurements. Model-based methods are the only viable way to solve the passive multi-target tracking problem, since the full fingerprinting approach cannot be used in practical multi-user scenarios due to the expensive calibration steps.

## 6. Conclusions

In this paper, we discuss the problem of passive localization and tracking of two moving targets in an area covered by a wireless mesh network. The key topic of the paper is the design and assessment of a dual-target physical–statistical model based on the diffraction theory to describe the fading induced by the two targets and infer their position by a Bayesian particle filter (PF) algorithm. In addition to the PF method, the successive cancellation (SC), the joint maximum likelihood (JML) and the radio tomographic maximum likelihood (RTI-ML) methods have been designed, implemented and compared to estimate the locations of the moving targets by exploiting both RSS average and variance information. Numerical results, obtained by an experimental indoor campaign, show that the proposed PF Bayesian algorithm with the diffraction-based model can achieve an average localization accuracy of about 0.6 m without requiring an extensive calibration phase as mandated by other multi-target fingerprinting methods. The proposed method proves to be a promising and viable solution for multi-target tracking in DFL systems.

## Figures and Tables

**Table 1:** Parameters adopted for the PF algorithms. (${}^{*}$) Note: only 7 landmarks out of 63 are used for model calibration (see Section 5.2).

Parameter	Additive Fingerprinting (AF)	Diffraction Model (DM)
*N*	18	18
*L*	306	306
*K*	2	2
*M*	63	63 (${}^{*}$)
*T* [ms]	360	360
$N_{p}$	600	600
$\sigma_{x}$ [m/s${}^{2}$]	0.77	0.77
$\sigma_{y}$ [m/s${}^{2}$]	0.77	0.77
Ω	25	25
δ	0.1	0.1
*B* [cm]	—	20
$a_{1},a_{2}$ [cm]	—	41
$b_{1},b_{2}$ [cm]	—	12
$H_{1},H_{2}$ [cm]	—	150
*h* [cm]	—	70

**Table 2:** RMSE [m] of localization for different algorithms.

Parameter	Additive Fingerprinting (AF)	Diffraction Model (DM)
PF	0.31	0.61
JML	0.61	0.89
SC	0.68	1.18
RTI-ML	0.51	1.05
RTI-LS	0.78	1.11

## Data Availability

Data sharing not applicable.

## Abbreviations

The following abbreviations are used in this manuscript:

2D	two-dimensional
3D	three-dimensional
CQI	Channel Quality Information
DFL	Device-Free Localization
EM	Electromagnetic
JML	Joint Maximum Likelihood
LOS	Line-Of-Sight
MAC	Media Access Control
ML	Maximum Likelihood
MMSE	Minimum Mean Squared Error
pdf	probability density function
PF	Particle Filter
PHY	PHYsical
RF	Radio Frequency
RSS	Received Signal Strength
RTI	Radio Tomographic Imaging
RX	Receiver
SoC	System-on-Chip
SC	Successive Cancellation
TX	Transmitter
